# Survival in Patients With De Novo Metastatic Prostate Cancer

**DOI:** 10.1001/jamanetworkopen.2024.1970

**Published:** 2024-03-12

**Authors:** Martin W. Schoen, R. Bruce Montgomery, Lukas Owens, Saira Khan, Kristen M. Sanfilippo, Ruth B. Etzioni

**Affiliations:** 1 Saint Louis Veterans Affairs Medical Center, Saint Louis, Missouri; 2 Saint Louis University School of Medicine, Saint Louis, Missouri; 3 VA Puget Sound Healthcare System, Washington; 4 Fred Hutchinson Cancer Center, Seattle, Washington; 5 University of Washington School of Medicine, Seattle; 6 Washington University in St Louis School of Medicine, Saint Louis, Missouri

## Abstract

This cross-sectional study investigates trends in overall survival among patients with newly diagnosed metastatic prostate cancer in 2 national registries in the United States.

## Introduction

Overall survival (OS) in metastatic hormone-sensitive prostate cancer (mHSPC) has improved in clinical trials over the last 20 years.^1,2^ It is unclear whether new treatments have translated to improvements in survival rates in clinical practice. We sought to quantify trends in OS among patients with newly diagnosed de novo (synchronous) mHSPC in 2 national registries in the United States: the Surveillance, Epidemiology, and End Results 17 (SEER) registry database and the Veterans Health Administration (VHA) registry database.

## Methods

This cross-sectional study was approved by the St Louis Veterans Affairs institutional review board and granted a waiver of informed consent due to its retrospective nature. Patients diagnosed from 2000 to 2019 were included if stage at first diagnosis of prostate cancer was distant metastasis from SEER or VHA Oncology registry. Patients in VHA were observed until death or April 2022. Cox proportional hazard modeling was used to assess mortality risk by age and calendar interval.

This study was conducted according to the STROBE reporting guideline. Statistical analysis was performed using SAS version 9.4 (SAS Institute) from July to September 2022. Two-sided *P* < .05 was considered statistically significant.

## Results

This study included 58 859 patients who were identified in SEER (median [IQR] age, 72 [64-81] years) and 14 904 patients in VHA (median [IQR] age, 73 [65-81] years). From the years 2000 to 2004 to the years 2015 to 2019, median OS increased from 23.0 to 30.0 months in SEER (hazard ratio [HR], 0.80 [95% CI, 0.77-0.82]); median OS increased from 25.6 to 30.9 months in VHA (HR, 0.91 [95% CI, 0.87-0.96]) (Figure). Among patients younger than 70 years, median OS increased from 31.0 to 40.0 months (HR, 0.78 [95% CI, 0.74-0.82]) in SEER and 34.3 to 42.2 months (HR, 0.87 [95% CI, 0.80-0.95]) in VHA. Among patients aged 70 years or older, increases were more modest; from 19.0 to 24.0 months in SEER (HR, 0.83 [95% CI, 0.80-0.86]) and from 21.6 to 25.6 months in VHA (HR, 0.94 [95% CI, 0.88-1.00]).

**Figure.**
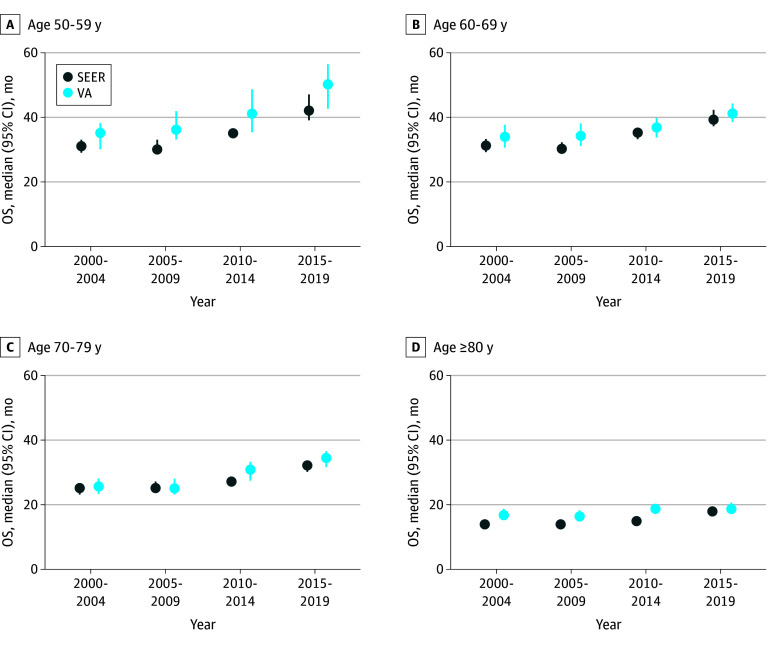
Median Overall Survival (OS) of De Novo Metastatic Prostate Cancer in the Surveillance, Epidemiology, and End Results 17 (SEER) and Veterans Health Administration (VHA) Registries The figure shows median survival by year of diagnosis, stratified by age at diagnosis of de novo metastatic prostate cancer in SEER and VHA registries. Error bars represent 95% CIs of the median.

## Discussion

This cross-sectional study found that median OS in de novo mHSPC was similar in SEER and VHA and had improved significantly in the US population from 2000 to 2019, particularly in patients younger than 70 years. The improvements in OS mirror those observed in other countries^3^ and are likely due to the increased use of combination therapy.^4^ Our results suggest that new treatment paradigms may contribute to longer OS in clinical practice, which is the ideal setting to measure improvements in outcomes as treatments change.

Increased OS from trials cannot be assumed to reflect improvements in disease management in clinical practice. The OS of men with mHSPC is lower in clinical practice than in clinical trials as patients are typically older with more comorbidities. Second, trials that test similar clinical scenarios can produce drastically disparate OS. For example, the median control group OS was 36.5 months in LATITUDE,^2^ which is shorter than the median control group OS of 70.2 months in SWOG-S1216,^1^ even though both trials had similar control groups and started enrollment in 2013.

Our results highlight the poor OS in patients over 80 years of age, with little improvement over time. This finding emphasizes the need to understand toxic effects vs benefits and competing risks for mortality in older patients. Older patients with comorbid disease may not benefit from new therapies and may have higher risk of adverse events. Tailoring treatment for mHSPC based on comorbidities^5^ or genetic features^6^ may improve patient outcomes. This study is limited by lack of comorbidity data; furthermore, because it is observational, causality cannot be determined.

With improved detection of mHSPC, we expect that more patients will be determined to have mHSPC at diagnosis with smaller burden of disease and with longer survival due to stage migration rather than from improvements in treatment. Separating the effects of treatment from the effects of earlier mHSPC diagnosis will be more complex in the future.

## Notes

Supplement.
Data Sharing Statement
