# Supplementary material for: Survival in Patients With De Novo Metastatic Prostate Cancer
Source: JAMA Netw Open. 2024 Mar 12;7(3):e241970. doi: 10.1001/jamanetworkopen.2024.1970 (PMC10936110; doi:10.1001/jamanetworkopen.2024.1970)
Supplement: Supplement. — Data Sharing Statement [file jamanetwopen-e241970-s001.pdf]

Data Sharing Statement

Schoen. Survival in Patients With De Novo Metastatic Prostate Cancer. *JAMA Netw Open*. Published March 12, 2024. doi:10.1001/jamanetworkopen.2024.1970

Data
Data available: Yes

Additional Information
Explanation for why data not available: The data from SEER is publically available. Data from the VA is available upon request to the first author with a data use agreement.
